# Effects of a carbohydrate-electrolyte solution on cognitive performance following exercise-induced hyperthermia in humans

**DOI:** 10.1186/s12970-014-0051-x

**Published:** 2014-11-04

**Authors:** Jason KW Lee, Wee Hon Ang, Jonathan WX Ng, Priscilla WP Fan, Ya Shi Teo, Heinrich W Nolte, Yvonne YW Yeo

**Affiliations:** Combat Protection and Performance Programme, Defence Medical and Environmental Research Institute, DSO National Laboratories, Singapore, Republic of Singapore; Department of Physiology, National University of Singapore, Singapore, Republic of Singapore; Lee Kong Chian School of Medicine, Nanyang Technological University, Singapore, Republic of Singapore; ERGOnomics TECHnologies, Research and Development, Armscor, Pretoria, South Africa

## Abstract

**Background:**

There is limited information on the effects of sports drinks on cognitive function after exercise in the heat. We aimed to investigate the effects of ingesting a commercially available carbohydrate-electrolyte (CHO) solution on cognitive performance following exercise-induced hyperthermia.

**Methods:**

Twelve participants completed three practices of cognitive tests, one full familiarisation and two experimental trials in an environmental chamber (dry bulb temperature: 30.2 ± 0.3°C, relative humidity: 70 ± 3%). The experimental trials consisted of five cognitive tests (symbol digit matching, search and memory, digit span, choice reaction time and psychomotor vigilance test) performed before and after a 75-min run on a treadmill at 70% VO_2_ max. One ml/kg body mass of a 6.8% CHO solution or placebo was consumed at the start, every 15 min during exercise and between cognitive tests after exercise. Core temperature, heart rate, blood glucose concentrations, subjective ratings and cognitive performance were assessed (symbol digit matching, search and memory, digit span, choice reaction time and psychomotor vigilance).

**Results:**

Participants were hyperthermic at the end of the run (placebo: 39.5 ± 0.4°C, CHO: 39.6 ± 0.5°C; Mean ± SD; p = 0.37). The change in blood glucose was higher with CHO ingestion (1.6, 0.7 to 4.5 mmol/L) (median, range) than with placebo ingestion (0.9, -0.1 to 4.7 mmol/L; p < 0.05). CHO ingestion reduced the maximum span of digits memorized, in contrast to an increase in maximum span with placebo ingestion (p < 0.05). CHO solution had no effect on other cognitive tests (p > 0.05).

**Conclusions:**

These results suggest that CHO solution ingestion may impair short-term memory following exertional heat stress.

## Background

Diminished cognitive ability is of concern to a wide variety of people who are routinely engaged in physical activity under heat stress. Examples of such occupations include professional athletes, soldiers, and civil defense personnel. Cognitive performance has been shown to degrade following exercise-induced hyperthermia [1]. Bursill [2] studied the effects of exertional hyperthermia on attention in 18 heat-acclimatised individuals. He observed that a funneling of peripheral awareness towards the central field of vision occurred in a high proportion of participants and inhibited their ability to respond appropriately to the presented stimuli. In a more recent study, Morley et al. [3] investigated the effect of treadmill exercise while wearing thermal protective clothing on cognitive function. While cognitive performance did not change immediately after exercise, short term memory was reduced 60 and 120 min after exercise. Furthermore, the mean of the 10 slowest reaction times increased 120 min after exercise. It was noteworthy that while 50 min of treadmill exercise in thermal protective clothing resulted in near maximal physiological strain, alterations in neurocognitive performance were not observable until an hour or more following exercise.

There are several possible reasons explaining impaired cognitive function following exercise-induced heat stress. These include dehydration [4], competition for attentional resources [5], and the availability of blood glucose to power cognitive processes [6]. All cellular activities in the body require energy in the form of ATP to sustain, including the brain cells. Most of the ATPs used by the brain are derived from the oxygen-dependent metabolism of glucose. Since the brain is the most metabolically active organ in the body, it is particularly vulnerable to the disruption of energy resources. In addition, it is also noteworthy that the brain possesses paradoxically limited stores of glycogen, which without replenishment are exhausted in up to 10 min [7]. Depletion of ATP at the cellular level can cause failure of membrane ion-transport systems due to neuron depolarization. Therefore, neural dysfunction due to inadequate blood glucose may be one important underlying mechanism of cognitive impairment following exercise-induced hyperthermia.

The role of glucose in the modulation of cognitive processes is well established. Beneficial effects of glucose supplementation have been observed across different populations under various experimental conditions. For example, it has been demonstrated that glucose supplementation can enhance learning and memory in healthy young and aged humans [8,9]. Enhanced cognitive performance caused by elevated plasma glucose levels has also been reported in schizophrenia patients [10]. Improved glycemic control was shown to enhance performance on selective areas of cognition [7]. Although glucose has beneficial effects on a range of cognitive tasks, evidence from the literature suggests that glucose has the most prominent effects on memory performance [7].

To our knowledge, there is limited information available on the effects of sports drinks on cognitive function after exercise in the heat [11]. The objective of this study was to investigate the effects of ingesting sports drinks on cognition following exertional heat stress. We hypothesized that the ingestion of sports drinks, as compared with a placebo, will alleviate the degree of cognitive impairment following exercise-induced hyperthermia.

## Methods

Twelve male endurance athletes (age: 23.8 ± 1.7 years; VO_2_ max: 59.4 ± 5.3 mL/kg/min) participated in this study that was approved by the DSO Institutional Review Board. Following an informed consent session explaining the nature, benefits and risks of the study, participants gave their consent verbally and in writing. Participants were certified fit for participation in this study by a physician. Before the commencement of each trial, participants completed a health status questionnaire to ensure that they were well.

Each participant visited the laboratory on five separate sessions. The first two sessions were conducted to familiarise participants with the cognitive test battery. Anthropometric measurements and peak aerobic capacity (VO_2_ max) were also determined during the first and second sessions respectively. Participants completed a full familiarisation trial (exercise and cognitive test battery) in the third session prior to the experimental trials with either the carbohydrate-electrolyte (CHO) solution or placebo in a counterbalanced manner.

Height was measured to the nearest 0.1 cm using a stadiometer (Seca, Brooklyn, N.Y., USA) and body mass was obtained to the nearest 0.05 kg using a digital scale (ID1 Plus/KCC150, Mettler-Toledo, Germany) during the first session. Skin-fold measurements were taken at four sites (biceps, triceps, subscapular and suprailiac) using skin-fold calipers (Model HSK-BI-3; Baty International, West Sussex, UK). Body density [12] and percent body fat [13] were calculated.

Peak aerobic capacity was measured during the second session. Each participant ran at four different speeds on a treadmill (h/p/cosmos Mercury, Germany), starting at a speed 1 km/h slower than his expected pace for a 10 km race, with increments of 1 km/h every 3 min, for a total of 12 min. Heart rate (HR) and ratings of perceived exertion (RPE) were recorded during the last 10 s of each 3 min stage. Oxygen uptake (VO_2_) was assessed over the final min of each 3 min stage through a mouthpiece connected to a metabolic cart (Cortex Metalyser 3B, Germany). Following the 12 min-run, participants were given 5 – 10 min rest before they started on a continuous treadmill run with an initial gradient of 1%, which increased by 1% every one min until volitional exhaustion [14]. Heart rate was measured continuously by short-range telemetry using a HR monitor (Model S810i; Polar Electro, Oy, Kempelem, Finland). Maximum oxygen uptake (VO_2_ max) was recorded as the mean oxygen uptake over the last min before volitional exhaustion. The relationship between VO_2_ and speed was determined and the speed corresponding to 70% VO_2_ max for each participant was used in subsequent experimental trials.

**Figure 1:**
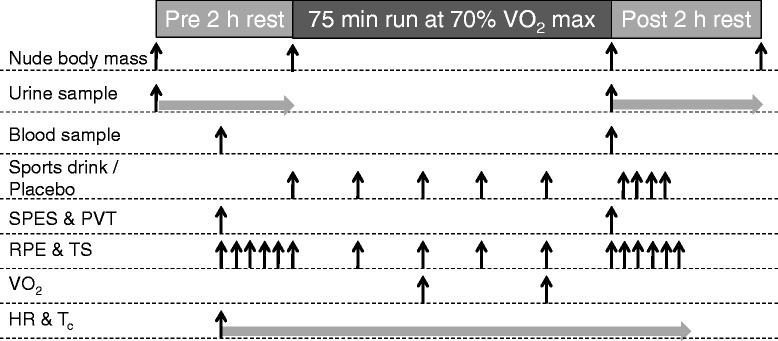
**Experimental protocol.**

Trials were separated by at least seven days to allow recovery. Participants were required to standardize their dietary intake, as well as to refrain from alcohol consumption and strenuous activity 24 hours prior to each trial. A dietary record sheet was provided to aid them in standardising their dietary intake. Each participant ingested a Jonah temperature capsule (Mini Mitter Co., Inc., Bend, OR, USA) at least 8 hours prior to the commencement of trial as a measurement of core temperature (*T*_*c*_). They were also requested to consume 500 mL of water 90 min before the commencement of each trial and refrain from drinking thereafter. A schematic of the experimental protocol is shown in Figure 1.

Upon arrival at the laboratory for each experimental trial, urine sample was collected before measurement of nude body mass. Urine osmolality was measured using freezing point depression (Osmomat 030-D, Gonotec, Germany). The VitalSense® temperature logger (Mini Mitter Co. Inc., Bend, OR, USA) was sealed in a waterproof bag fitted into a padded pouch, and worn on a customised lightweight harness around the waist positioned in the lumbar region of the participant. Each participant also wore a chest band and wrist watch HR monitor. A climatic monitoring device (Squirrel 2020 data logger, Grant Instruments, United Kingdom) was used to measure dry bulb temperature and relative humidity during the experimental trials.

Participants entered an environmental chamber (Heraeus Votsch, Germany) programmed to maintain a dry bulb temperature of 30°C and 70% relative humidity. A baseline blood sample was obtained after participants stood for 10 min. Participants commenced with the Swedish Performance Evaluation System (SPES) [15] that assessed a variety of cognitive functions such as symbol digit matching, search and memory, digit span, choice reaction time, followed by the Psychomotor Vigilance Test (PVT) [16]. The total duration of the cognitive test battery took approximately 40 min. The test battery was performed before and immediately after exercise in the environmental chamber.

Participants performed a run at 70% VO_2_ max for 75 min on a motorized treadmill following completion of the baseline cognitive test battery. Participants were required to consume 1 ml/kg body mass of sports drinks (100Plus, F&N, Singapore; CHO trial) or placebo (control) before the start, every 15 min during, immediately after exercise and in between the cognitive tests after exercise. The nutritional compositions of the sports drink and placebo are shown in Table 1. Ratings of perceived exertion and thermal sensation (RTS) were assessed before the start, every 15 min during exercise, at the beginning and the end of each cognitive test. Trials were terminated prematurely when a participant’s core temperature reached 40°C, in adherence with pre-determined ethical guidelines. Following completion of the 75 min run, blood sampling, body mass measurements and urine sampling were performed before participants completed the post-exercise cognitive test battery. Sweat loss was estimated from the difference in body masses, corrected for fluid intake and urine production: (pre-exercise body mass – post-exercise body mass) + ingested fluid – urine output.

**Table 1:** **Energy content and composition of placebo and sports drink**

**Drink contents**	**Placebo**	**Sports drink**
Energy value (kcal/L)*	6	270
Fat (g/L)	0	0
Protein (g/L)	0	0
Carbohydrate (g/L)	0	68
Sucrose (g/L)	0	48
Glucose (g/L)	0	20
Osmolality (mOsmol/kg)*	29 ± 1	349 ± 11
Sodium (mmol/L)	0	18 ± 3
Potassium (mmol/L)	0	3 ± 0

*There may be trace amounts of sugars present in the placebo drink which could not be detected by our analysis, thus explaining the energy and osmolality values despite the absence of carbohydrates.

Venous blood samples (4.5 mL each) were collected from the participants’ forearm after they stood for 10 min and immediately after the run. The blood was dispensed into an anticoagulant-free tube and blood glucose concentrations were assessed using a commercial glucose analyser (Acc-Chek® Advantage, Roche, Germany).

The Statistical Package for Social Sciences version 15.0 was used to perform all statistical computations. Normality of experimental trial data was assessed using the Shapiro-Wilk test. Paired t-test was performed on data that were normally distributed. Data that did not follow the normal distribution were analyzed using Wilcoxon matched-pair signed rank test. A 5% level of significance was used for all statistical analyses. Results with normal distribution were reported in mean and standard deviation while those that did not follow a normal distribution were reported in median and range.

## Results

### Environmental conditions and pre-trial hydration status

There were no differences in dry bulb temperature (placebo: 30.2 ± 0.3°C vs. CHO: 30.2 ± 0.3°C; p = 0.86) and relative humidity (placebo: 72 ± 2% vs. CHO: 69 ± 3%; p = 0.10) between the placebo and CHO trials. Participants were considered euhydrated prior to each trial as indicated by their urine osmolality (placebo: 163, 74 to 669 mOsmol/kg [median, range]; CHO: 205, 102 to 621 mOsmol/kg; p = 0.41).

### Exercise intensity and duration during run

Participants ran at similar exercise intensity with placebo (70 ± 5% VO_2_ max) and CHO ingestion (71 ± 5% VO_2_ max, p = 0.12). Eight subjects terminated exercise early during one or both experimental trials. Out of these eight subjects, two terminated exercise early as their core temperature has reached the approved ethical limit of 40°C. Six subjects stopped running before 75 min due to exhaustion. Statistical analysis revealed similar exercise duration between the placebo (67 ± 8 min) and the CHO trials (66 ± 13 min, p = 0.73). Removal of these participants from the analysis of cognitive, physiological and blood data had no effect on the interpretation of results, hence their data were included.

### Core temperature

No difference was found in core temperature between the placebo and CHO trials at the start of the run (placebo: 36.7 ± 0.2°C vs. CHO: 36.7 ± 0.2°C; p = 0.79), at end of the run (placebo: 39.5 ± 0.4°C vs. CHO: 39.6 ± 0.5°C; p = 0.37), at the beginning of the post-exercise cognitive test battery (placebo: 38.9 ± 0.4°C vs. CHO: 38.8 ± 0.5°C; p = 0.93), and at the end of the post-exercise cognitive test battery (placebo: 37.3 ± 0.3°C vs. CHO: 37.4 ± 0.5°C; p = 0.37).

### Heart rate

Heart rate was similar between the placebo and CHO trials at the start of the run (placebo: 58 ± 7 bpm vs. CHO: 59 ± 7 bpm; p = 0.19), at end of the run (placebo: 171 ± 10 vs. CHO: 171 ± 10 bpm; p = 0.97), at the beginning of the post-exercise cognitive test battery (placebo: 99 ± 16 bpm vs. CHO: 98 ± 14 bpm; p = 0.73), and the end of the post-exercise cognitive test battery (placebo: 82 ± 10 bpm vs. CHO: 85 ± 10 bpm; p = 0.14).

### Ratings of thermal sensation (RTS) and perceived exertion (RPE)

No difference was found in mean RTS between the placebo (4.1 ± 0.9) and CHO trials (4.1 ± 0.8; p = 0.49). Mean RPE was also similar between the placebo (13 ± 4) and CHO trials (13 ± 4; p = 0.08).

### Blood glucose

Comparing the placebo and the CHO trials, the intra-subject increase in blood glucose (pre-exercise to post-exercise) was higher with CHO ingestion (1.6, 0.7 to 4.5 mmol/L [median, range]) than with placebo ingestion (0.9, -0.1 to 4.7 mmol/L; p < 0.05).

### Sweat rate and fluid balance

Sweat rate was similar between the placebo (1.77 ± 0.47 L/h) and CHO trials (1.72 ± 0.35 L/h, p = 0.83). Body mass loss during the run was similar between the placebo (1.4 ± 0.6 kg) and CHO trials (1.4 ± 0.5 kg; p = 0.94).

### Cognitive tests

**Figure 2:**
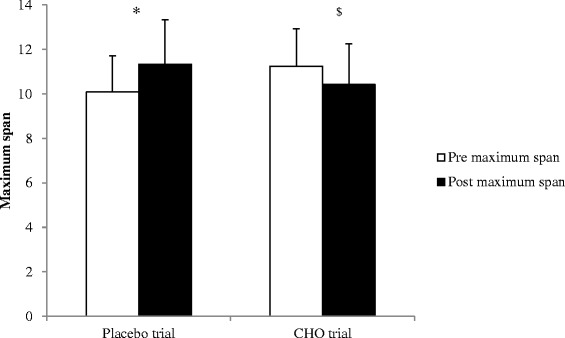
**Effect of placebo and CHO ingestion on short-term memory (n = 12).** * indicates increased in maximum span of digits memorized from pre- to post-exercise for the placebo trial (p < 0.05). ^$^ indicates decreased in maximum span of digits memorized from pre- to post-exercise for the CHO trial (p < 0.05).

Results of the cognitive tests are shown in Table 2. Test scores from the symbol digit matching, search and memory, choice reaction time, and PVT showed no differences between the placebo and CHO trials (p > 0.05). CHO ingestion reduced the maximum span of digits memorized in the digit span test (p < 0.05), in contrast to an increase in maximum span with placebo ingestion (p < 0.05; Figure 2). Out of our 12 participants, only one performed better for the digit span test during the CHO trial while performance was similar between trials in three other participants.

**Table 2:** **Cognitive test summary (n = 12)**

**Cognitive test**	**Measures**	**Pre-placebo**	**Post-placebo**	**Change**	**Pre-CHO**	**Post-CHO**	**Change**
Symbol Digit Matching	Mean Reaction Time (ms)	1624 ± 279	1490 ± 232	-134 ± 154	1693 ± 265	1525 ± 276	-169 ± 139
	No. of Errors	2 ± 3	2 ± 2	0 ± 4	2 ± 2	2 ± 3	0 ± 3
Search & Memory	Mean Reaction Time (ms)	4437 ± 1661	4311 ± 1309	-126 ± 807	4464 ± 1666	4411 ± 1707	-53 ± 603
Level 1	No. of Errors	0 ± 1	1 ± 1	0 ± 1	1 ± 1	1 ± 1	0 ± 1
Search & Memory	Mean Reaction Time (ms)	6984 ± 2406	6693 ± 3331	-291 ± 1955	7256 ± 2552	6862 ± 2782	-393 ± 1007
Level 2	No. of Errors	1 ± 1	2 ± 2	1 ± 2	2 ± 1	2 ± 2	1 ± 2
Search & Memory	Mean Reaction Time (ms)	9711 ± 3799	9384 ± 4314	-328 ± 2077	9936 ± 3658	9317 ± 4381	-619 ± 2337
Level 3	No. of Errors	1 ± 2	1 ± 2	0 ± 1	3 ± 2	3 ± 2	0 ± 3
Digit Span	Maximum Span	10 ± 2	11 ± 2	1 ± 2	11 ± 2	10 ± 2	-1 ± 1
	No. of Directional Changes	8 ± 2	9 ± 2	1 ± 3	8 ± 1	9 ± 2	1 ± 2
Choice Reaction Time	Number of Correct Responses	121 ± 6	120 ± 15	-1 ± 13	122 ± 5	122 ± 9	0 ± 6
	Mean Reaction Time (ms)	574 ± 88	522 ± 71	-51 ± 84	562 ± 74	546 ± 96	-16 ± 57
Psychomotor Vigilance	Median Reaction Time (ms)	262 ± 55	244 ± 46	-18 ± 30	254 ± 42	236 ± 47	-18 ± 19
	No. of Errors	3 ± 3	1 ± 2	-2 ± 3	2 ± 2	2 ± 4	0 ± 4

## Discussion

This study assessed the efficacy of ingesting a commercially available carbohydrate-electrolyte solution (CHO) on cognitive performance following exercise-induced hyperthermia. Prior to the main experimental trials, all participants underwent three practices of the cognitive test battery to minimize learning effect. The study indicated that ingestion of CHO solution resulted in a decrease in the longest string of digits memorized in the digit span test, but had no effect on the performance of the other cognitive tests (symbol digit matching, search and memory, choice reaction time and PVT). This suggests that CHO ingestion led to a degradation in short-term memory after a 75 min run in the heat but had no effect on perceptual capacity and speed, executive control functioning, speed of decision making, vigilance, and response latency. There are several possible reasons why ingestion of a CHO drink would impair short-term memory, including glycemic index and rapid swings in blood glucose levels.

The rate of glucose delivery depends on physicochemical properties of the food, which can be summarized by the glycemic index (GI), a parameter that reflects the rate and extent of the postprandial rise and fall in blood glucose [17]. Glucose, which was ingested by our participants during the CHO trials, is a carbohydrate with high GI. This is significant as high GI foods can induce rapid changes in blood glucose levels, which have been associated with fatigue and reduced cognitive function [18,19]. Furthermore, there is evidence to support the notion that foods with lower glycemic loads are associated with better performances on aspects of cognitive functioning [6,20,21]. A study conducted by Nilsson et al. [21] examined the effects of GI on cognitive performance. In that study, a white wheat bread enriched with guar gum was able to produce a low but sustained blood glucose net increment. Working memory and selective attention were subsequently evaluated with this white wheat bread enriched with guar gum in the postprandial period against a high GI white wheat bread only. It was found that cognitive performance was improved in participants who ingested the white wheat bread enriched with guar gum. Similarly, Benton et al. [17] also showed that consuming a high GI meal resulted in poorer memory performance when compared to a low GI meal in both humans and rats. The influence of the GI of breakfast on verbal memory of adults was measured throughout the morning and it was found that a low GI diet (vs. a high GI diet) improved memory, especially in the late morning (150 and 210 min after breakfast). After 3 h following breakfast, it was found that rats displayed better learning performance after they were fed with a low GI diet.

The interaction between blood glucose and memory function has been shown to follow an inverted U-shaped dose-response curve; best memory performance was observed when increase in blood glucose was between 0.6 – 2.8 mmol/L [22]. Although the rise in blood glucose levels following CHO ingestion (1.6, 0.7 to 4.5 mmol/L) was within the optimum range for memory performance, it was unknown if this increase persisted throughout the entire cognitive test battery. Further increase in blood glucose levels caused by ingesting more CHO solution following exercise could have stimulated a higher insulin response in our study. A sharper profile of postprandial insulin release induced by high GI diets is likely to result in more glucose being directed towards insulin-sensitive tissues such as muscles, liver and white adipose tissue. As a consequence to the insulin surge, blood glucose levels may decrease which can lead to disruption of glucose delivery to the brain, thus slowing down local energy recruitment in the brain when cognitive demand occurs [17,21]. Since blood glucose was only analyzed immediately after exercise in this study, we do not know the glucose level during the actual cognitive tests.

The lack of significant effect of CHO ingestion on the other cognitive tests in this study could be due to the cognitive test battery being not demanding enough for the participants. Studies have suggested that ingestion of carbohydrate is more likely to improve cognitive function of higher complexity [23-25]. Glucose ingestion was found to enhance verbal episodic memory performance in studies that employed a divided attention protocol [26,27], but not in studies lacking in this protocol [28-30]. The divided attention protocol involved participants performing a secondary task during encoding of a supraspan memory list. The difficulty of cognitive test is also relative to the age of the participant as glucose was found to have greater enhancing effects in older adults than younger adults, and also in tasks with high levels of difficulty or that require divided attention [6,7,23]. This may be related to the notion that healthy young adults are operating at their ‘cognitive peak’; therefore, a cognitive enhancer would only be effective when individuals experienced an increase in cognitive demands [31,32]. Since participants in our study were mainly young adults (age: 23.8 ± 1.7 years), one possible reason for the lack of cognitive benefits after CHO could be due to low task difficulty.

Participants in our placebo group experienced an increase in blood glucose levels after exercise (0.9, -0.1 to 4.7 mmol/L). During exercise, plasma glucagon is known to increase in response to the decreasing blood glucose and insulin levels [33]. Glucagon acts on the liver to break down glycogen stores and release glucose into the bloodstream, maintaining blood glucose levels during exercise. The increased blood glucose levels after exercise observed in this study could be due to a spillover effect of the increased plasma glucagon induced by exercise. This increased blood glucose level may be sufficient to allow the placebo group to handle the difficulty of the cognitive tests, thus achieving similar results as the CHO group. Additional intake of glucose (as seen in the CHO group) may be unnecessary for the brain during the cognitive tests, and may even have detrimental effects on cognitive performances due to high insulin response as discussed above.

## Conclusions

In conclusion, these results suggest that CHO solution ingestion may impair short-term memory following exertional heat stress but does not have any effect on working memory, executive control function, speed of decision making and vigilance. Commercially available sports drink may thus offer negligible cognitive benefits.
